# The extents of metabolic syndrome among Antiretroviral Therapy exposed and ART naïve adult HIV patients in the Gedeo-zone, Southern-Ethiopia: a comparative cross-sectional study

**DOI:** 10.1186/s13690-020-00420-3

**Published:** 2020-05-07

**Authors:** Girma Tenkolu Bune, Alemayehu Worku Yalew, Abera Kumie

**Affiliations:** 1 School of Public Health, Dilla University, Dilla, Ethiopia; 2 Schools of Public Health, Addis Ababa University/AAU, Addis Ababa, Ethiopia

**Keywords:** Metabolic syndrome, Human immunodeficiency virus, People living with HIV, Antiretroviral therapy (ART) exposed, ART naïve

## Abstract

**Background:**

HIV infection and Antiretroviral Therapy (ART) has been associated with metabolic syndrome (MS). The prevalence of MS varies substantially between populations and is not yet well-known in sub-Saharan Africa (SSA), including Ethiopia. The current study aims to estimate and evaluate the magnitude of MS among ART exposed and ART naïve HIV-infected patients.

**Methods:**

A comparative cross-sectional design was employed among the randomly chosen PLHIVs from two hospitals and two health centers, found in the Gedeo zone, southern-Ethiopia. Data collection was run beginning from December 29th, 2017 up to January 22nd − 2019, using the WHO steep tool; eventually, the completed data entered into Epidata (V-3.1) and exported to SPSS (V^− 22^) for analysis. The revised international diabetes federation criterion was used to define MS and its components. The mean, standard deviations and proportions were used as a descriptive summary. Categorical data and the proportion of MS in the two groups were compared using binary logistic regression, and results were reported statistically significant with *p*-value is less than 5%.

**Results:**

A total of 633 (*n* = 422 on ART and *n* = 211 ART-naive) PLHIVS was involved, with an overall response rate of 96.2%. The cumulative proportion of MS was 42.5%(95% CI: 39.2–45.7), with 43.4%(95% CI: 39.1–47.4) among ART exposed and 40.8% (95% CI: 35.5–46.0) among ART naïve patients (*P* > 0.005). However, the difference was not statistically significant and signified that ART has no association with an increased proportion of MS.

**Conclusion:**

Overall this study demonstrated the presence of an elevated degree of overall MS among PLHIVs. Besides, although the difference was not statistically significant, a relatively higher proportion of MS was realized in the ART exposed than ART naïve groups. Implicated that at the time of the entire test and treatment approaches employed in this target group, routine screening of MS incorporated through HIV care and management system will be a vibrant action.

## Background

While global efforts are integrated into the combat against HIV, the prevalence of people living with HIV (PLHIVs) constantly increases due to the rollout of antiretroviral therapy (ART) [1]. In 2018, 23.3 million PLHIVs were accessing ART up from 7.7 million in 2010. As of the end of June 2019, 24.5 million people living with HIV (PLHIVs) were accessing the treatment [2]. This access was complemented with a 51% reduction in HIV mortality, from 1•95 million in 2006 to 0•95 million in 2017 [3], improved quality of life and survived longer [4, 5].

With increased survival, the global morbidity and mortality from infectious diseases have occupied a backseat [6]; instead, non-AIDS comorbidity, like a non-communicable disease (NCDs), following acquiring its risk marker known as Metabolic Syndrome (MS) has become a worldwide public health issue [6–8]. MS is commonly defined as a constellation of interconnected complex diseases such as abdominal fat, high blood pressure, dyslipidemia, elevated blood sugar [5, 9, 10]. Though not identical, there have been diverse standardized definitions used to diagnose MS [10], each with criteria that influence its diagnosis and complexity [6, 8, 11–14].

Although much effort on the provision of chronic care has been focused on the control of opportunistic infections [1], not as much care is on the relationship between HIV infection, ART and MS development among PLHIVs [5]. This syndrome was initially recognized in HIV patients a few years after the initiation of ART [6]. Since then, it has imposed countless challenges to the world health designers due to its consequences in various lifestyle diseases [6]. Adult individuals with MS will have 3-times likely to have Cardiovascular Diseases (CVDs) and a 5-fold greater risk of acquiring Type Two Diabetes Mellitus (T2DM) [9, 15–18]. They are non-AIDs associated causes of morbidity and mortality in PLHIVs [1, 4]. For this reason, presently, it is regarded as a newly re-emerging challenge of the PLHIVs population [9, 19], in addition to achieving the 90: 90: 90 targets and moving to ‘test and treat’ [19].

Several works of literature have shown that the overall prevalence of MS risk among PLHIVs is inconsistent and is high [5, 11, 14], with ranges of 16.7–31.3% [10]. As well, a narrative review from Africa and a meta-study from the sub-Saharan Africa (SSA) countries reported comparably higher ranges of (13–58%) (9, 15.1–26.9%) [5] of MS in PLHIVs, respectively.

Moreover, studies also pinpointed that the mechanisms of MS and its traits among PLHIVs are well predicted and attributed to the HIV infection and the ARV regimen [1, 5, 11]. The MS change begins early in the course of HIV infection [7, 8]. The inflammatory response and cellular apoptosis are the two mechanisms associated with MS development and HIV infection [1]. Even after ART initiation, the number of cytokines (which is a pro-inflammatory agent) in the blood remains higher [6] and the impact on the components of MS is to be more pronounced [6, 7]. Overall, the adverse effect of ART causes dyslipidemia, lipodystrophy, and mitochondrial dysfunction side effects, which are implied to initiate body mechanisms that lead to the occurrence of MS [1].

Despite these facts, yet, little is known about the extents of MS and the difference of burden among PLHIVs with or without ART in SSA [5, 14, 20]. Much of studies from resource-rich World primarily noted MS burden among all PLHIVs, regardless of their ART status [5, 10, 21, 22]. Besides, such studies in the same setting are inadequate [14]; particularly, in the era of test and treat and increasing survival due to the treatment effect and epidemiological shifts taking place in the region [5, 14]. Whether ART exposed to ART naïve individuals have a high risk of MS remains debatable.

Thus, the current study aims to estimate and evaluate the magnitude of MS among ART exposed and ART naïve individuals, by using the revised International Diabetes Federation (IDF) criteria [12]. This manuscript is an extension of a large study with many objectives (of which one article was published [13]). The former and the latter criteria used by the manuscripts are different in various aspects; perhaps, reflecting the dynamic nature of the methods resulted from the aggregate facts of the subject’s gathered over time [6, 8, 12, 13].

The study is significant to Ethiopia, including SSA that allows a great dual load following the long-drawn-out epidemiological change. It is a paramount research urgency area, as relevant primary data are limited, particularly in the era of growing access to treatment in the region, including the study country and zone, where the issue has not yet been well addressed, to the best of our knowledge. Besides, initiating such a study has a paramount significance to pave the way to tackle the syndrome among PLHIVs, once for all, in the countries of East African as a whole and Ethiopia, in particular, where they lack contextually developed specific criteria and fully dependent on that standard criterion formed in the western mindset.

## Methods and materials

### Study design

A comparative crossectional study approach was used.

### Study contexts

Gedeo zone was the place where this study was conducted. It is located 360KM to the south of Addis Ababa, the main city of Ethiopia. The study was run beginning from December 29th, 2017 to January 22nd, 2019. During the study period, as per the Gedio zone ART case team Health Management Information system (HMIS) report, a total of 3597 adult PLHIVs (629 ART-naive (370 female, 259 male) and 2968 currently on ART (1813 female, 1155 male)) existed in the zone. Of which, while 2922 (1790 female and 1132 male) of them were in the first-line regimen, only 46 (23 female, 23 male) were in the second-line regimen. Among these subjects, while 1892 (135 ART naïve and 537 ART exposed) were enrolled in the ART clinics of health centers, the remaining 2807 (412 ART naïve and 2395 ART exposed) of them were enrolled in the hospitals found in the zone [13]. As per the currently revised national guideline for HIV prevention, Care and Treatment protocol, not all people living with HIV are eligible for ART and have got access to ART immediately [29]. With regards to the ART regimens, it is possible to refer to the guideline cited and the pre-published segments of this article with this citation [13].

### Participants

**Fig. 1:**
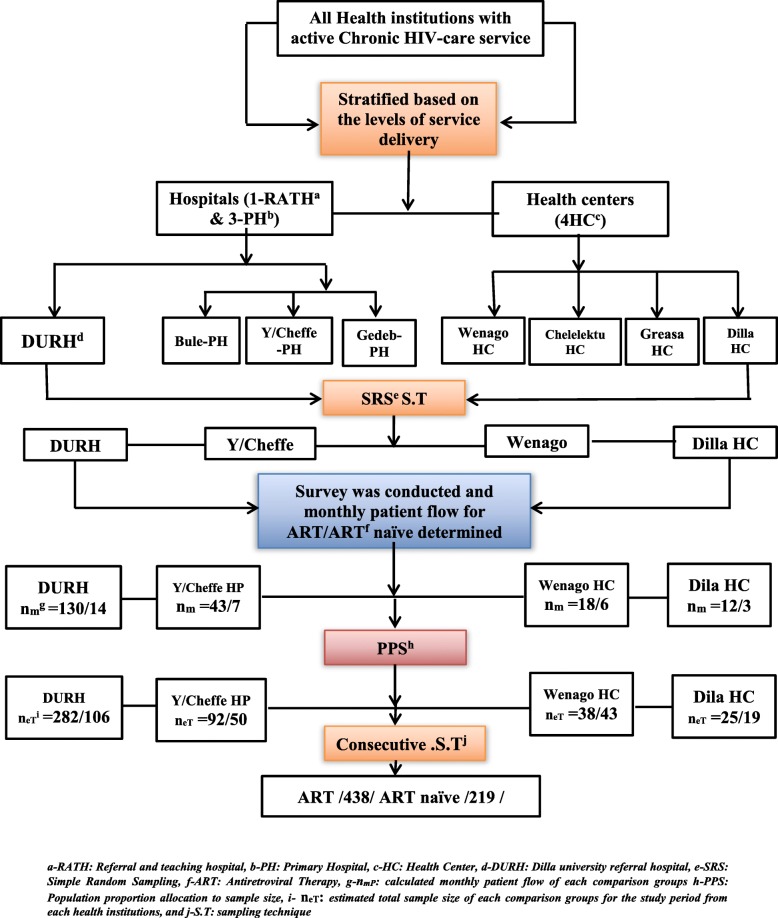
The schematic representation of the study procedure, among PLHIVs, in the Gedeo zone, Southern-Ethiopia, 2017–2019

The study was conducted among PLHIVs took routine service under randomly selected two hospitals and health centers found in the zone. Before recruiting the participants, a survey accompanied by the data logs at the data clerk of the clinics of each health care institution was arranged for a month, to assess the daily/weekly/monthly patient flow. Using the monthly assessment result, with the employment proportional allocation of a sample size to both comparative groups (ART-exposed and ART naïve), the necessary cases to each health institution for 1 year period was predicted. Lastly, the daily enrollment of participants based on the eligibility criteria was undergone using a consecutive sampling method (Fig. 1).

### Sample size determination

The sample size estimation was made using Openepi version 3.03 software and by taking the following assumptions into account: the two population proportion identified from the previous study done in Hawassa university specialized Hospital, Sothern Ethiopia [23], (P1 = 15.6% for ART naïve and assuming 13% difference (i.e. 18.1% among ART-exposed), with 1:2 ratio of the ART naïve and ART-exposed groups, 95% confidence interval, 5% level of significance, and with the power of 90%, the calculated sample size for the ART naïve groups became 199. By adding 10% non-response, overall 657 (n1 = 219 and n2 = 438) HIV positive patients were proposed for the study.

### Data collection procedure

The data collection was accomplished using the validated WHO STEPS instrument version 3.2[30]. It covers three different levels or ‘steps’ of risk factor assessment: Step 1 (questionnaire), which was essential to gather demographic and behavioral characteristics of the PLHIVs. Step 2 (physical measurements), was employed to build on the core data in step 1 and to determine the proportion of the participants with raised blood pressure, overweight and obesity. The NUTEC BP09 Arm-type Fully Automatic Digital BP Monitor was used to measure blood pressure three times on the left arm of the study participants. A constant tension tape meter was used to measure waist circumference (WC). The 220 SECA scale was used to measure body height and weight, and then the result was used to calculate BMI. Step 3(Biochemical measurements) was used to measure the percentage of respondents with impaired fasting plasma glucose, diabetes, and abnormal lipid level. Laboratory investigation was done sometime after completion of STEPS 1 and 2 of the data collection process, with 8–12 h overnight fast, by withdrawing of 5 mL blood. The BS-200E Clinical Chemistry Analyzer was used to analyze biochemical measures to all samples serum collected centrally at Dilla University referral and teaching hospital’s clinical Diagnostic Laboratory unit. While the enzymatic colorimetric assay method was used for the measurement of all lipid profiles (i.e. total triglyceride and High-density lipoprotein cholesterol), the glucose level was measured by using the glucose oxidase method.

### Outcome variables definition

Globally there exists a diverse internationally recognized standard criterion to diagnose overall MS [5, 10, 13, 17, 23, 24]. This study was defined using the revised International Diabetes Federation (IDF) criterion [12]. It is the most commonly applied criteria to diagnose the cumulative burden of MS among PLHIVs [12]. Based on the new IDF criteria, for a person to be defined as having the condition, he /she must have central obesity (i.e. evaluated from either elevated waist circumference with ethnicity-specific values (men/women > 94 cm/80 cm) or body mass index >/=30 kg/m^2^) plus any two of the following four factors: raised triglycerides (≥150 mg/dL (1.7 mmol/L) or specific treatment for this lipid abnormality); reduced high-density lipoprotein cholesterol (< 40 mg/dL (1.03 mmol/L) in males < 50 mg/dL (1.29 mmol/L) in females or specific treatment for this lipid abnormality); raised blood pressure (BP) (systolic BP ≥130 or diastolic BP ≥85 mmHg or treatment of previously diagnosed hypertension), and raised fasting plasma glucose (FPG) ≥100 mg/dL (5.6 mmol/L), or previously diagnosed with diabetes [12].

### Data processing and analysis

Often, after completion of each step of the data collection, the completeness of the tools was checked by the immediate supervisor and the main author. The completed tools entered into a template formed using Epidata version3.1 software by two data clerk; eventually, validation was performed using the original data as references. Subsequently, data were transformed into Statistical Package for Social Sciences (SPSS) Version 22 for analysis. Statistical analysis was performed by the principal investigator in consultation with the primary and secondary supervisors. Charts were produced using Microsoft® Excel 2007. Depending on the scale of the variable the mean, standard deviations, and proportions were presented as a descriptive summary. Categorical data and the magnitude of MS in the ART exposed and naïve groups were compared using a binary logistic regression, and results were reported statistically significant whenever the *p*-value is less than 5%.

In light of this, for the rest of the methodological procedure, you can refer to the published paper elsewhere [13].

### Ethical clearance

All the ethical guidelines and principles placed in the Declaration of Helsinki and others, necessary to address the ethical aspects of the research initiated in humans were taken into account. Based on that, the proposal submitted to Addis Ababa University (AAU) College of Health Sciences School of public health Research and Ethics Committee (REC) and then to College of health science Institutional Review Board (IRB) (Meeting No.001/2017 and protocol No.0069/16/SPH) to obtain ethical clearance. Subsequently, the official letter granted from the School of public health by citing the above ethical approval reference number was distributed to the respective Southern Nations Nationalities Regional health bureaus, Gedio zone, and Woreda health bureaus, including to all of the institutions selected to conduct the study. Lastly, to ensure voluntary participation, written consent preceded with oral was attained from each individual.

## Result

### The socio-demographic characteristics

Overall, 633 (*n* = 211 ART-naive and *n* = 422 on ART exposed) individuals were participated in the study, with the response rate of 96.2%. More than half (64.1%) (69.0% ART exposed and 54.5% ART naïve) of the participants were residents of the urban area, and 59.4% (376) of them were women, with the average age of (36.4 ± 8.7) years old. Among the 302 participants who were capable of reminding the duration of living with HIV after diagnosis, higher percentage 43.9% (278) ((58.3% ART naïve and 36.7% ART-exposed) of them were reacted that it was lower than a year, with the average duration of (5.31 ± 3.99) months. Moreover, the result revealed that 53.5%(226) of women and 46.5% (196) of men were on either the 1st and 2nd line ART regimen. Of which, nearly 98 of the participant with ART were recalling and guess the time duration since they initiated. Eight point 4 % (7.0% of men and 9.3% of women) of these participants were reported to be exposed for about 6 months and below, with the mean reported duration of 5.69 ± 3.00 months (6.27 ± 3.09 months for men and 5.31 ± 2.90 months for women).

### Physical and biochemical measurements

The mean and standard deviation for each of the physical and biochemical criterion measurements were computed, and the table below summarized the finding as follows (Table 1).

**Table 1:** The mean and standard deviation (SD) of physical and biochemical measurements, by ART status, among PLHIVS, in the Gedeo zone, Southern-Ethiopia, 2017–2019

**S.No**	**Criteria**	**ART status**				**Total**	
		ART^a^ naïve^b^(*n* = 211)		ART exposed(*n* = 422)			
		Mean	SD	Mean	SD	Mean	SD
1	Waist circumference (WC)	83.7	8.2	83.5	7.7	83.6	7.9
2	Body Mass Index (BMI)	22.22	4.36	22.18	4.54	22.19	4.48
3	Systolic blood pressure (SBP)	126.9	7.8	126.9	11.5	126.9	10.4
4	Diastolic (DBP)	82.8	5.1	83.3	6.3	83.1	5.9
5	High-density lipoprotein (HDL_c)	52.8	23.3	58.9	23.8	56.8	23.8
6	Triglycerides (TGL_c)	133.5	30.2	145.9	32.3	141.8	32.1
7	Fasting plasma glucose (FPG)	102.2	30.1	112.6	39.1	109.1	36.7
9	Total cholesterol (TC_c)	168.9	41.0	181.5	45.2	177.3	44.2
10	Low-density lipoprotein (LDL_c)	138.4	32.7	146.6	31.9	143.9	32.4

*SD Standard deviation, ^a^-ART: Antiretroviral Therapy, ^b^-naïve: ART unexposed

**Fig. 2:**
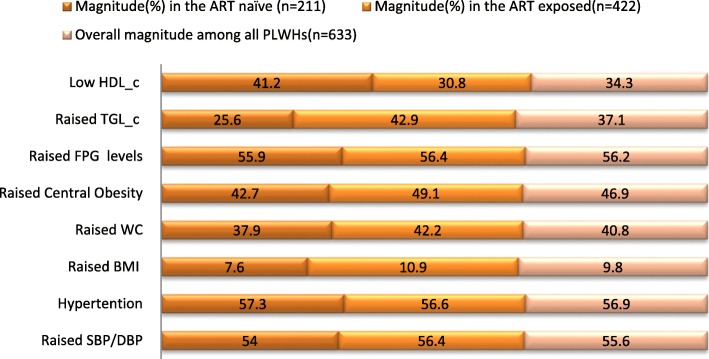
Magnitudes of each physical and biochemical measures, aggregated by ART status, among PLHIV, in the Gedeo-zone, southern Ethiopia, 2017–2019

Inline, the study further determined the magnitude of each of the physical and biochemical measured parameters. All in all, the finding designated the presence of an elevated level of each criterion in the ART groups than ART naïve, except for the case of BMI and HDL_c. On top of that, excluding for the case of TGL_c and HDL_c, overall the result from the binary logistic regression represented the presence of insignificant differences across the two comparison groups(*P* > 0.005) (Table 2), and (Fig. 2).

**Table 2:** Magnitudes of physical and biochemical measures, aggregated by ART status, among PLHIV, in the Gedeo-zone, southern-Ethiopia, 2017–2019

**S.No**	**The IDF parameter**	**Magnitude(%) by ART status**				**Total**		***P*-value**
		ART naïve (*n* = 211)		ART exposed(*n* = 422)				
		%	95% CI	%	95% CI	%	95% CI	
1	Raised SBP/DBP>/=130/85 mmHg^a^	54.0	47.4–60.2	56.4	52.1–61.1	55.6	51.8–59.6	0.57
2	Raised blood pressure (BP) (SBP/DBP or Anti-HTN treatment user)	57.3	1.0–1.11	56.6	1.0–1.07	56.9	1.03–1.07	0.87
3	Raised BMI >/= 30 kg/M^2b^	7.6	4.3–10.9	10.9	8.1–13.7	9.8	7.6–12.2	0.19
4	Raised WC (WC > Women 80 or men > 94 cm)	37.9	31.3–44.1	42.2	37.7–47.2	40.8	37.1–44.5	0.30
5	Central Obesity (CO) (WC or BMI)	42.7	37.4–49.3	49.1	44.5–53.3	46.9	43.3–50.7	0.13
7	FPG (FPG > 100 mg/dl^c^ or Anti-DM treatment user)	55.9	49.3–62.5	56.4	52.1–61.1	56.2	52.6–60.0	0.91
8	Raised TGL_c (TGL_c>/=150 mg/dl or treated for TGL_c)	25.6	19.9–31.3	42.9	37.9–47.4	37.1	33.0–40.8	< 0.001
9	Low HDL_c (Women< 50 mg/dl or Men < 40 mg/dl or treated for TGL_c)	41.2	34.6–47.4	30.8	26.3–35.5	34.3	30.6–38.1	0.009

^a^-mmHg: millimeter of mercury, ^b^-Kg/M^*2*^: Kilogram per square meter, ^c^-mg/dl: milligram per deciliter.

### The magnitude of metabolic syndrome (%MS)

**Fig. 3:**
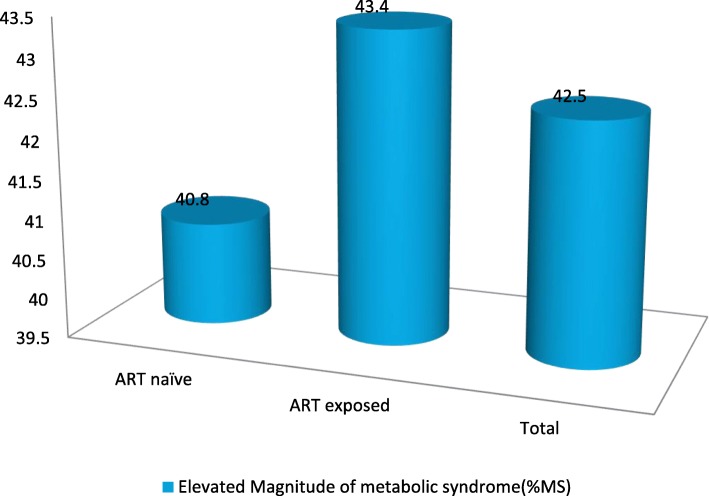
The magnitude of metabolic syndrome (MS) by ART status, in the Gedeo zone, southern-Ethiopia, 2017–2019

The overall proportion of MS was about 42% (42.5, 95% CI: 39.2–45.7), with a relatively higher differences being realized in the ART exposed (43.4, 95% CI, 39.1–47.4) than ART naïve (40.8, 95% CI, 35.5–46.0) groups (*p* > 0.005) **(**Fig. 3). However, the observed difference seen across groups was not statistically significant **(*P*** **> 0.005) (**Table 3**).**

**Table 3:** Magnitude of metabolic syndrome (%MS), among PLHIVSs, in the Gedeo-zone, southern-Ethiopia, 2017–2019

**S.no**	**Factors**	**n**	**Elevated Magnitude of metabolic syndrome(%MS**^**a**^**)**		***P*-value**
			**%(95% CI)**^**b**^	**OR(95%CI)**^**c**^	
1	**ART status**	633			
	ART naïve	211	40.8 (35.5–46.0)	1	
	ART exposed	422	43.4 (39.1–47.4)	1.3 (0.9–1.9)	.247
2	**Age groups in years**	633			
	</= 34	315	24.4 (19.4–28.9)	1	
	35–44	203	54.7 (48.3–61.1)	4.6 (3.0–7.1)	< 0.001
	>/= 45	115	70.4 (61.7–78.3)	8.4 (5.0–14.2)	< 0.001
2	**Sex**	633			
	Men	257	21.8 (16.7–26.8)	1	
	Women	376	56.6 (52.1–61.2)	5.24 (3.5–7.8)	.000
3	**Residents**	633			
	Urban	406	46.8 (41.9–51.5)	1	
	Rural	227	34.8 (28.6–41.4)	0.7 (0.5–1.0)	.078
	Total	633	42.5 (39.2–45.7)		

a-%MS: Magnitude of Metabolic syndrome b-%(95% CI): Proportion and its 95% confidence intervals. c-OR (95%CI): Odd ratio and 95% confidence intervals

**Fig. 4:**
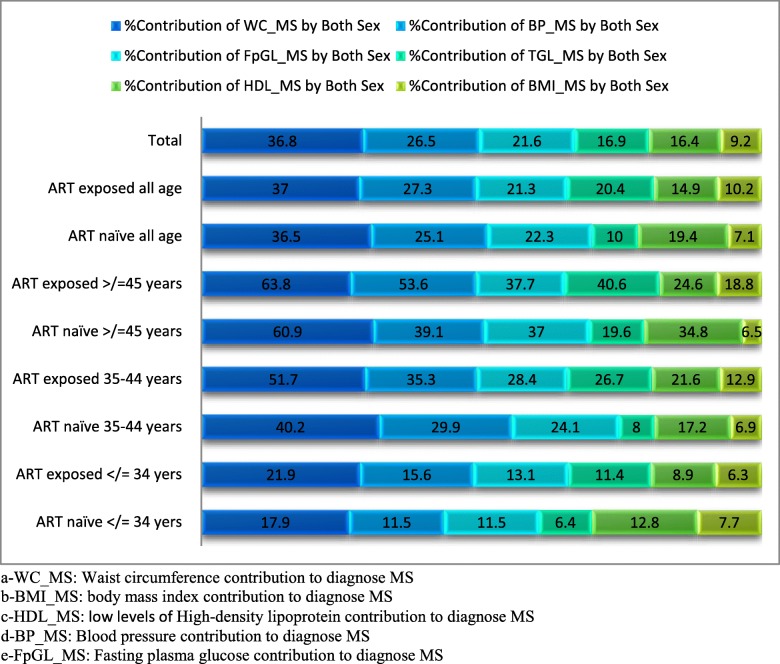
the percentage contribution of physical and biochemical measurements to diagnose MS, among PLWH, in the Gedeo-zone, Southern- Ethiopia, 2017–2019

On top of that, for the descriptive purposes, this study furthermore assessed the percentage contribution of each of the parameters in the diagnosis of the burden of MS in both groups and was summarized in Fig. 4 as follows **(**Fig. 4).

## Discussion

Overall, this study demonstrated the presence of an elevated degree of MS among PLHIVs. Even if the differences were not significant, a relatively higher proportion of MS was realized in the ART exposed (43.4%) than ART naïve (40.8%) counter groups. Furthermore, the study revealed that elevated WC contributed to the highest proportion, followed by elevated blood pressure measures, impaired fasting plasma glucose, raised triglycerides, low HDL_c and body mass index.

In contrast to our finding, a narrative review from different African countries by Husain et al. (2017) and the study from Southwestern Uganda by Muyanja et al. (2016) were reported relatively higher magnitude of MS renges (13–58%) 9 [9, 25]. A recent crossectional study from Ethiopia by G.T. Bune et al. (2019) (22.0, 95% CI: 19.0–25.4)) [13], by Hirgo.et al. (2016) (24.3%) [20], by Tesfaye et al. (2014) (25%) [21] and Berhane T, et al. (2012) (21.1%) [26], a meta-study from the SSA, by Todowede et al. (2019) (21.5, 95%CI: 15.09–26.86)) [5] and by Nguyen KA, et al. (2016) worldwide (24.6–31.3%) [10] were reported lower proportion of MS burden among PLHIVs as a whole, regardless of their ART status. The difference might be partly associated with the difference of sample size used, the overlapping of the host, the virus and/or the type of ART regimen related differences [5, 7, 10, 17, 20].

Further, our study has highlighted the presence of a higher magnitude of MS in the ART exposed groups than the ART naïve; however, the observed difference was not statistically significant, (*P* > 0.005). Analogs with the finding, a narrative review in the globe designated the presence of differences of extents of MS among ART exposed and unexposed individuals, with a relatively higher range of (18.4–21.6% ART exposed vs.11.8–19.9% ART naïve) MS magnitude [5, 7, 10, 17, 20]. Specifically, for example, a study by G.T. Bune et al. (2019) (22.5, 95% CI: 18.7–26.8 ART exposed vs. 20.9, 95% CI: 15.2–27.1 ART naïve, *P* > 0.05) [13], a meta-analysis study in the globe (18.4, 95%CI: 15.9–21.1 in the ART-exposed and 11.8, 95%CI: 9.3–14.7, in the ART-naïve *p* = 0.001) [10] were also revealed same. However, the proportion reported to both groups was inconsistent and was below the reported levels of the present study. Regardless of the risk of differences of MS among the two groups in our study result not being significant, it is key that the magnitude of MS in both comparative subjects is raising and ranged in the estimated level of the SSA region. This denotes that the extent of MS is on the rise intensely in PLHIVs with or without ART in the study zone. Implicated that extra factors such as aging, diets, and other lifestyles, associated factors require vital attention other than HIV infection and ART [5].

Also, in contrary to our result, a study from Cameroon by Ngatchou W, et al. (2013) was presented a lower rate of MS in the ART-exposed (21%), and an extremely higher magnitude of MS in the ART naïve groups (47%) [27]. The time variation, the number of study participants, the differences in study approaches, and the socio-economic and cultural factors associated with the host could be partially credited for the above differences observed by the studies.

In addition to the above reasoning, the discrepancy may be partly attributed to the various criteria employed by the studies [5]. With this regard, the recent study by G.T. Bune et al. (2019) [13] can be taken as a typical example. As it has been presented elsewhere, the data source for the current and the previously published article was the same. However, the results were inconsistent, and this might be explained due to the difference in standard criteria employed by the studies. Similarly, empirical evidence from several works of literature was unpacking facts that support the above concept. In this regards, for example, a meta-study by Nguyen KA, et al. (2016) was shown distinction of MS burden among similar groups based on the difference of standard criteria used (i.e. in the ART-exposed, by (IDF criteria 19.6, 95%CI: 14.2–25.6 vs. the ATP III criteria 21.6, 95%CI: 13.5–31.0 14.9%), and in the ART-naïve, by (IDF criteria 14.9, 95%CI: 8.6–22.6 vs. the ATP III criteria 19.9, 95%CI: 18.3–21.5) [10]. Moreover, a study by Tesfaye et al. (2014) shown variations (in the ART exposed, by (IDF criteria 25% vs. ATP III 18.1%) and in the ART naïve, by (IDF, 22.6 vs. ATP III 15.6%) [21]. As it is presented in the literature above, even if there were no reliable differences of MS estimates in between the IDF and ATP III criteria. However, the overall situation underscored the presence of a higher estimation of MS by the IDF criteria in both ART exposed and ART nave groups. This is comparable with our study finding that higher estimates based on the IDF criteria. This indicates a greater waist circumference (WC) in both exposed and unexposed groups enrolled in the study, particularly women, as this is a mandatory condition by IDF criteria [5, 20]. It was comparable with the report highlighting elevated central obesity based on the WC and BMI score in PLHIVs individuals, particularly in the ART exposed group [20]. This implicated the need for further study to explore the dissimilarity in WC based on ART status. Moreover, it also implicates the critical significance of establishing harmonized standard criteria, which is to be generated in the East-African countries’ contexts in general and in the Ethiopia perspective, in particular, to resolve the problem arises from the use of the available standard criterion constructed in the western mindset.

Last, of all, this study has to have several limitations, all of which were inherited from the nature of crossectional study design, institution-based studies, and interviewer assisted data collection methods used. This study also not free from the social desirability bias, and recall biases arose from the individual seeking further medical care, and from inquiring of participants about past experiences, respectively. Besides, it has limitations related to the consideration of relatively fewer men and ART-naïve participants, and the absence of an HIV-negative sub-groups that might potentially affect external validity was the finding.

## Conclusion

Generally, this study demonstrated the presence of an elevated degree of MS among PLHIVs. Also, even if the diffrence was not significant, a relatively higher proportion of MS was realized in the ART exposed than ART naïve groups. This signifies the existence of HIV associated MS that necessitates immediate prevention and management strategies in such a resource-restricted area. On top of that, at the time of the entire test and treatment plan of action among HIV infected patients, routine follow-up of MS encompassed through the whole management system is to be a vibrant action.

## Data Availability

All data generated or analyzed during this study are included in this published article [and its supplementary information files].

## Abbreviations

AIDS: Acquired Immunodeficiency syndrome
ART: Antiretroviral Therap
CVD: Cardio Vascular Disorder
HDL_c: High-Density Lipoprotein Cholesterol
IRB: Institutional Review Board
LDL-c: Low-Density Lipoprotein Cholesterol.
MS: Metabolic Syndrome
NCDs: Non-communicable disease
NCEP/ATP-III: National cholesterol Education program adult treatment panel three
NRTI: Nucleosides reverse transcriptase inhibitors
NNRTI: Non-Nucleosides Reverse Transcriptase Inhibitors
PI: Protease inhibitors
PLHIVS: People Living with HIV
3TC: Lamuvidine
ABC: Abacavir
AZT/ZDV: Zidovudine
EFV: Efavirenz
LPV/r: Ritonavir boosted/ Lopenavir
NFV: Nelfinavir
NVP: Nevirapine
DDI: Didanosine
ATV/r: Ritonavir boosted/ Atazanavir
DRV/r: Ritonavir-boosted/ Darunavir
